# Recombinant Klotho attenuates IFNγ receptor signaling and SAMHD1 expression through blocking NF-κB translocation in glomerular mesangial cells

**DOI:** 10.7150/ijms.78279

**Published:** 2023-04-29

**Authors:** Kuen-Daw Tsai, Yi-Chao Lee, Bo-Yu Chen, Li-Syuan Wu, Shan-Yuan Liang, Ming-Yuan Liu, Yu-Wen Hung, Hui-Ling Hsu, Pei-Qi Chen, Jia-Ching Shieh, Yi-Ju Lee, Ting-Hui Lin

**Affiliations:** 1 School of Biomedical Sciences, College of Medical Science and Technology, Chung Shan Medical University; Department of Medical Research, Chung Shan Medical University Hospital, 110 Jianguo North Road, Section 1, Taichung 40203, Taiwan, ROC.; 2 Department of Internal Medicine, China Medical University Beigang Hospital, 123 Sinde Road, Beigang Township, Yunlin County, 65152, Taiwan, ROC.; 3 School of Chinese Medicine, College of Chinese Medicine, China Medical University, 91 Hsueh-Shih Road, Taichung, 40402, Taiwan, ROC.; 4 Ph.D. Program in Medical Neuroscience, College of Medical Science and Technology, Taipei Medical University, Taipei, 11031, Taiwan.; 5 Department of Respiratory Therapy, China Medical University Beigang Hospital, 123 Sinde Road, Beigang Township, Yunlin County, 65152, Taiwan, ROC.; 6 Institute of Medicine, Chung Shan Medical University, 110 Jianguo North Road, Section 1, Taichung 40203, Taiwan, ROC.

**Keywords:** IFNγ, SAMHD1, Klotho, glomerular mesangial cells, NFκB, JAK-STAT1

## Abstract

Interferon gamma (IFNγ) is a cytokine implicated in the pathogenesis of autoimmune diseases. SAM and HD domain-containing protein 1 (SAMHD1) is an IFNγ-inducible protein that modulates cellular dNTP levels. Mutations in the human *SAMHD1* gene cause Aicardi-Goutières (AG) syndrome, an autoimmune disease sharing similar clinical features with systemic lupus erythematosus (SLE). Klotho is an anti-inflammatory protein which suppresses aging through multiple mechanisms. Implication of Klotho in autoimmune response is identified in rheumatologic diseases such as SLE. Little information exists regarding the effect of Klotho in lupus nephritis, one of the prevalent symptoms of SLE. The present study verified the effect of IFNγ on SAMHD1 and Klotho expression in MES-13 glomerular mesangial cells, a special cell type in glomerulus that is critically involved in lupus nephritis. IFNγ upregulated SAMHD1 expression in MES-13 cells through the Janus kinase-signal transducer and activator of transcription 1 (JAK-STAT1) and the nuclear factor kappa B (NFκB) signaling pathways. IFNγ decreased Klotho protein expression in MES-13 cells. Treatment of MES-13 cells with recombinant Klotho protein inhibited SAMHD1 expression by blocking IFNγ-induced NFκB nuclear translocation, but showed no effect on JAK-STAT1 signaling. Collectively, our findings support the protective role of Klotho in attenuating lupus nephritis through the inhibition of IFNγ-induced SAMHD1 expression and IFNγ downstream signaling in MES-13 cells.

## Introduction

Interferon gamma (IFNγ) is the only type II interferon (IFN) produced by immune cells 1. IFNγ mediates inflammatory reactions and enhances immune responses by modulating interferome expression or priming the pathogen product. IFNγ contributes to autoimmune pathology and to systemic lupus erythematosus (SLE) in particular 2. SLE, commonly called lupus, is one type of autoimmune diseases characterized by immune dysfunction. Unusual nucleic acid metabolism associated with immune complexes formation may be one of the possible mechanisms that accounts for the production of multiple self-reactive antibodies seen in SLE patients 3. In SLE patients, multiple tissues and organs, especially kidneys, are attacked by immune system and cause injury. Clinically, about 50 to 75% of SLE patients develop lupus nephritis, a type of glomerulonephritis 4 which is the most critical symptom of SLE. In female NZB/W mice, which model the course of human SLE, mice eventually die of glomerulonephritis. The onset of glomerulonephritis was delayed when female NZB/W mice were treated with soluble IFNγ receptors or anti-IFNγ antibodies 5. Accordingly, blocking IFNγ downstream signaling is a potential therapeutic strategy for treating lupus nephritis 5.

SAM and HD domain-containing protein 1 (SAMHD1), first identified in mouse macrophages as an IFNγ-inducible protein 6, possesses both deoxynucleoside triphosphohydrolase (dNTPase) and exonuclease activity. SAMHD1 blocks viral replication by depleting cellular dNTP levels and participates in cell cycle regulation, nucleotide metabolism, and innate immunity 7. Mutations in *SAMHD1* gene is associated with Aicardi-Goutières (AG) syndrome, a hereditary encephalopathy characterized by disorders of immune response, especially over-activation of IFN production 8. Some patients with AG syndrome show clinically phenotypic features overlapping with infantile SLE 9-11. The responsiveness of SAMHD1 to inflammatory stimuli in different immune cells is controversial 12, 13. This discrepancy may be due to the complicated regulatory mechanisms of SAMHD1 gene expression. SAMHD1 was ubiquitously detectable in various tissues and expressed in cells of hematopoietic origin 13. In the kidneys, SAMHD1 was detected in glomerular mesangial cells which were derived from hematopoietic stem cells 14, 15 and was critically involved in glomerular injury such as lupus nephritis 16. Lupus nephritis is a major risk factor of mortality in SLE patients 17. Although the physiological function of SAMHD1 in kidney is unknown, SAMHD1 may be involved in lupus nephritis since mutations of *SAMHD1* gene have been identified in patients with AG syndrome, a disease clinically and genetically *overlap* with SLE 9. The mechanisms underlying the expression of SAMHD1 in mesangial cells have yet to be explored.

Klotho protein possesses anti-aging functions 18 and plays a role in multiple biological activities including anti-inflammation, removal of reactive oxygen species, and regulation of phosphate/calcium metabolism 19. Klotho is an important protein implicating in the pathogenesis of many human diseases. In models of human inflammatory bowel disease, renal expression of Klotho protein was significantly reduced 20. Predominant expression of Klotho protein in the kidneys was severely reduced in patients with chronic kidney disease 21. Decreased Klotho enzyme activities were observed in the CD4^+^ T lymphocytes of healthy older adults and in individuals with rheumatoid arthritis (RA) 22. Soluble Klotho protein was implicated as a potential biomarker in selected rheumatologic autoimmune diseases 23. Clinical studies showed decreased levels of Klotho in serum from patients with SLE 23. These reports indicated the relevance of Klotho in certain autoimmune diseases. Little information exists regarding the effect of Klotho in lupus nephritis.

Altogether, IFNγ 2, SAMHD1 8 and Klotho 23 have been implicated in autoimmune diseases. Both SAMHD1 6 and Klotho 20 were modulated by IFNγ, a major cytokine contributes to severity of SLE 2. Because children with AG syndrome develop clinically overlapping syndromes with SLE and lupus nephritis are major causes of mortality in SLE patients 4, these observations lead us to speculate the potential role of SAMHD1in lupus nephritis. MES-13 glomerular mesangial cells*, the major* contributor to renal damage seen in the *lupus nephritis* 16 were used in the present study. The central hypothesis to be tested is whether Klotho exerts a protective effect against renal inflammation caused by autoimmune responses, especially focusing on IFNγ receptor signaling and IFNγ-stimulated SAMHD1 expression. The major goal of the present study is to provide evidence that Klotho impeded inflammation in autoimmune responses. Our results demonstrated recombinant Klotho attenuates IFNγ receptor signaling and SAMHD1 expression through blocking NF-κB translocation in glomerular mesangial cells.

## Materials and Methods

### Materials

Fetal bovine serum was from Hyclone (Logan, UT). DMEM medium and Ham's F12 medium and medium supplements were obtained from Thermo Fisher Scientific Inc. (Waltham, MA). IFNγ (Cat. 315-05) was purchased from PeproTech EC Ltd. (London, UK). The specific antibodies for NF-kB (SC-8008), GAPDH (SC-32233), Histone H3 (SC-10809), anti-rabbit (SC-2357), anti-mouse (SC-2005) secondary antibodies for Western Blot were products from Santa Cruz Biotechnology (Santa Cruz, CA). The specific antibody for STAT-1(Cat. 603701) was purchased from BioLegend (San Diego, CA). The specific antibody for phospho-Stat1 (Tyr701) antibody (Cat. 9167) was purchased from Cell Signaling Technology Inc. (Beverly, MA). The antibody for SAMHD1 (ab128107) and Klotho (ab203576) and recombinant human Klotho protein (ab84072) were purchased from Abcam (Cambridge, MA). The fluorescein isothiocyanate (FITC)-conjugated secondary antibody [Alex Fluor® 488 AffiniPure Goat Anti-Mouse IgG (H+L), AB-2307324] for fluorescent microscopy was purchased from Jackson ImmunoResearch Laboratories Inc (West Grove, PA). STAT1 inhibitor (nifuroxazide) and NF-kB inhibitor ammonium pyrrolidinedithiocarbamate (PDTC) were obtained from Sigma Chemical Company (St. Louis, MO).

### Cell Culture

The MES-13 cell line was obtained from the American Type Culture Collection (CRL-1927; ATCC, Manassas, VA, USA) and maintained in a culture medium with a 3:1 mixture of Dulbecco's modified Eagle's medium and Ham's medium supplemented with 14 mM 4-(2-hydroxyethyl)piperazine-1-ethane-sulfonic acid (HEPES), 2 mM glutamine, antibiotics (100 μg/mL penicillin and 100 μg/mL streptomycin), and 5% fetal bovine serum at 37°C. The incubation chamber was equilibrated with 5% carbon dioxide and 95% oxygen. The passage numbers of MES-13 cells used were between 20 to 30 passages.

### Western Blot Analysis

To detect the protein levels of SAMHD1 and Klotho after exposure to different stimuli, MES-13 cells were washed with 1x PBS, scraped out, and incubated with lysis buffer. The lysis buffer contained 1x PBS, 1% NP-40, 0.5% sodium deoxycholate, 0.1% SDS and protease inhibitor cocktail. Cells suspended in lysis buffer were sonicated. The homogenate was centrifuged at 12,000 rpm for 30 min at 4^0^C, and the cell supernatant was collected. The protein concentration was measured using a Bio-Rad protein assay kit. Equal amounts of protein samples (20 μg) were subjected to sodium dodecyl sulfate polyacrylamide gel electrophoresis (SDS-PAGE). Following electrophoresis, the gel was transferred to a polyvinylidene difluoride membrane, blocked with 5% skim-milk in Tris-buffered saline (TBS) containing10 mM Tris (pH 8.0) and 150 mM NaCl, then incubated with primary antibody at 4^0^ C overnight. TBS containing 0.02% Tween 20 (TBST) was used to wash out the nonspecific binding material on the PVDF membrane. Finally, the membrane was incubated with secondary antibody for 1 h at room temperature. After washing with TBST, the immunoreactive bands were visualized with a light-emitting kit (ECL, PerkinElmer, MA, USA). The protein amount was quantified by measuring the area of the SAMHD1 or Klotho bands using densitometric analysis with AlphaEaseFC (Genetic Technologies, Miami, FL, USA) and normalized to glyceraldehyde 3-phosphate dehydrogenase (GAPDH). All experiments were performed at least in triplicate and reported as a percentage of the control.

### Preparation of Cytosolic and Nuclear Extracts

To determine the effect of IFN-γ, NF-κB inhibitor (PDTC) and recombinant Klotho protein on nuclear NF-κB translocation, cytoplasmic and nuclear proteins of MES-13 cells were prepared. MES-13 cells after exposure to different stimuli were washed with 1x PBS, scraped out, and incubated with lysis buffer on ice for 10 min. The lysis buffer contained 20 mM HEPES (pH 7.0), 10 mM KCl, 2 mM MgCl_2_, 0.5% Nonidet P-40 and protease inhibitor cocktail. Cells suspended in lysis buffer were homogenized by 30 strokes in a tightly fitting Dounce homogenizer, then centrifuged for 5 min at 3,000 rpm at 4^0^ C. The supernatant contains the cytoplasmic fraction and the pellet is nuclei fraction. The nuclear pellet was washed with lysis buffer and then resuspended in nuclear extract buffer for 30 minutes on ice with vortexing at 10-minute intervals. The nuclear extract buffer contained 50mM Tris, pH8.0, 150 mM NaCl, 2 mM EDTA, 1 % NP-40. The nuclear mixture was centrifuged at 13200 rpm for 20 min at 4°C. The supernatant is nuclear fraction. Activation of NF-κB in nuclear extracts was analyzed by Western blot.

### Fluorescent Microscopy (Immunocytochemical Staining)

To detect subcellular distribution of NF-κB, MES-13 cells were grown on coverslips and fixed in ice-cold methanol, followed by permeablization in 0.2% Triton X-100. Cells were incubated with blocking buffer (1% BSA) and then incubated with anti- NF-κB antibody, followed by FITC-conjugated Alex Fluor® 488 secondary antibody. Cells were counterstained with 4'-6-diamidino-2-phenylindole (DAPI) (Thermo Fisher Scientific Inc.) and mounted using p-phenylenediamine anti-fade mounting media. Visualization was performed using an Axioplan 2 Zeiss microscope and images were captured with a Carl Zeiss AxioCam system and analyzed by Axiovision Rel 4.8 software (Carl Zeiss).

### Statistical Analysis

All values are expressed as the mean ± SD and were derived from at least three independent experiments. Data were analyzed by using a one-way analysis of variance (ANOVA) followed by Dunnett method for multigroup comparison tests. A *p* value of <0.05 was considered statistically significant in all analyses. Data in this study was normally distributed.

## Results

### JAK-STAT1 pathway and NFκB activation were involved in IFNγ-induced SAMHD1 expression in MES-13 cells

**Figure 1:**
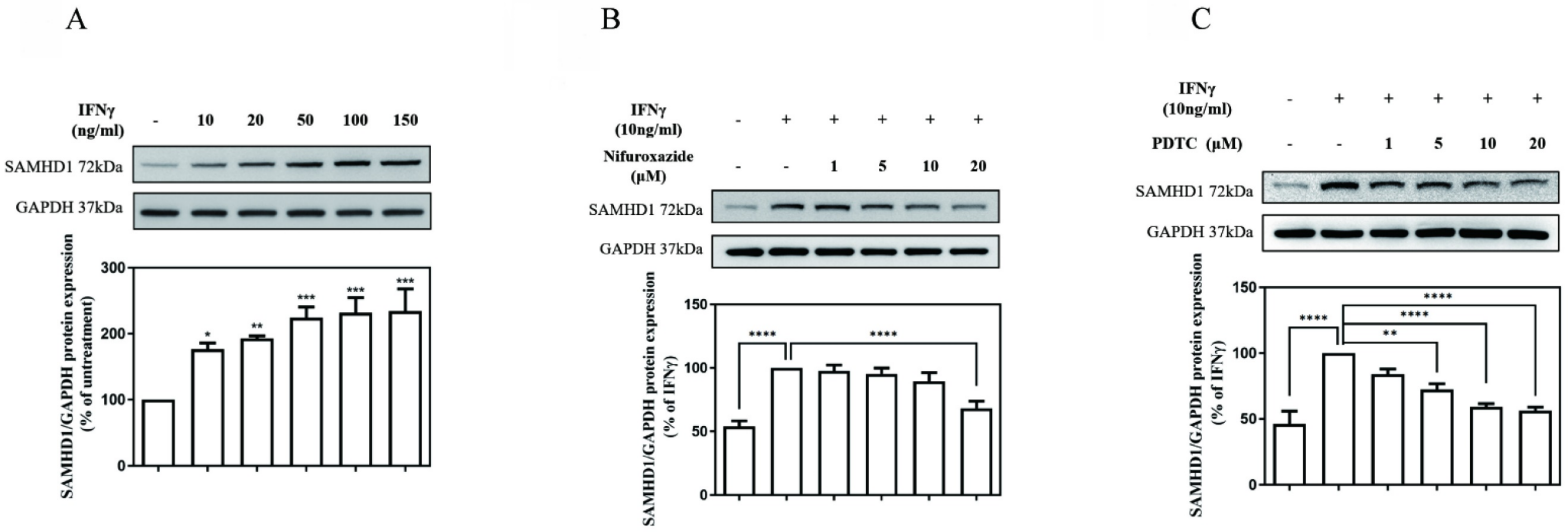
** JAK-STAT1 pathways and NFκB activation were involved in IFNγ-induced SAMHD1 expression in MES-13 cells.** In (A), MES-13 cells were treated for 24 h with different concentrations of IFNγ (10, 20, 50, 100, and 150 ng/mL), and SAMHD1 protein expression was determined through Western blot. The SAMHD1 protein level in untreated MES-13 cells was used as the control value and set to 100%. MES-13 cells were pretreated with either the STAT1 inhibitor nifuroxazide (1, 5, 10 and 20 μM) for 1 h (B) or with PDTC (1, 5, 10 and 20 μM), a NFκB inhibitor, for 30 min (C), followed by stimulation with 10 ng/mL IFNγ for 24 h. The SAMHD1 protein level in IFNγ (10 ng/mL)-treated MES-13 cells was used as the control value and set to 100%. GAPDH was used as an internal control. Relative protein levels were quantified through scanning densitometry, normalized to GAPDH and expressed as a percentage of the maximal band intensity in the control group. Data are presented as the mean ± SD of SAMHD1/GAPDH expression derived from at least three separate experiments. Asterisks indicate a significant difference from the control group (**∗*p* < 0.05, ∗∗*p* < 0.01, ∗∗∗*p* < 0.001**).

As illustrated in **Fig. 1 (A)**, the SAMHD1 protein level was markedly increased in cultures treated with IFNγ in a concentration-dependent manner. The value of the SAMHD1 protein level in IFNγ-untreated MES-13 cells was used as a control and set to 100%. The signals of the SAMHD1 protein increased substantially to176%, 192%, 224%, 231%, and 234% of the control value following treatment with 10, 20, 50, 100, and 150 ng/mL IFNγ, respectively. To investigate whether JAK-STAT1 signaling was involved in IFNγ-induced SAMHD1 expression, MES-13 cells were pretreated with the STAT1 inhibitor nifuroxazide (1, 5, 10, and 20 μM) for 1 h and then stimulated with 10 ng/mL IFNγ for 24 h. As illustrated in **Fig. 1 (B)**, the IFNγ-induced SAMHD1 protein level was significantly diminished to 68% of the control value after treatment with 20 μM nifuroxazide. These results indicated that JAK-STAT1 pathway was involved in IFNγ-induced SAMHD1 expression in MES-13 cells. As illustrated in **Fig.1 (C)**, MES-13 cells were pretreated with PDTC (a NFκB inhibitor) at 1, 5, 10 and 20 µM for 30 min, and then stimulated with 10 ng/mL IFNγ for 24 h. the IFNγ-induced SAMHD1 protein expression was significantly reduced to 83%, 72%, 59% and 56% of the control value. Therefore, IFNγ-induced SAMHD1 protein expression in MES-13 cells was not only regulated by JAK-STAT1 but also by NFκB signaling.

### IFNγ suppressed Klotho protein expression in MES-13 cells

**Figure 2:**
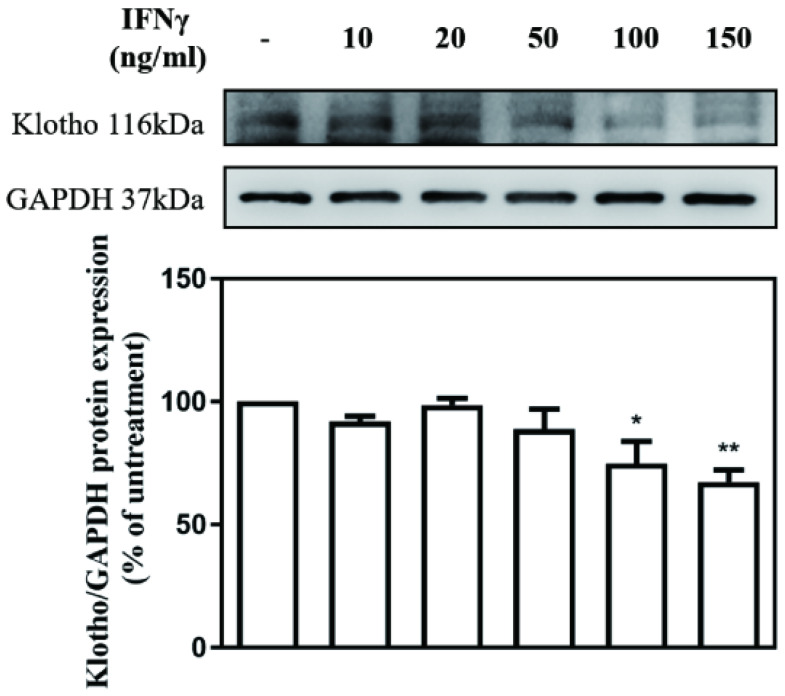
** IFNγ suppressed Klotho protein expression in MES-13 cells.** MES-13 cells were treated for 24 h with different concentrations of IFNγ (10, 20, 50, 100, and 150 ng/mL), and Klotho protein expression was determined by Western blot. GAPDH was used as an internal control. The relative protein levels were quantified through scanning densitometry, normalized to GAPDH and expressed as a percentage of the maximal band intensity of the Klotho protein from cultures treated without IFNγ. Data are presented as the mean ± SD of Klotho/GAPDH derived from at least three separate experiments. Asterisks indicate a significant difference from treatment without IFNγ (∗*p* < 0.05, ∗∗*p* < 0.01).

The effect of different concentrations of IFN-γ (10, 20, 50, 100, and 150 ng/mL) on Klotho expression in MES-13 cells was investigated. The value of Klotho protein level in IFNγ-untreated MES-13 cells was used as the control and set to 100%. As illustrated in **Fig. 2**, IFNγ in concentrations below 50 ng/mL exercised no effect on Klotho protein expression. As the concentration of IFNγ increased to 100 ng/mL and 150 ng/mL, the expression of Klotho protein decreased significantly to 74% and 67% of the control value, respectively. These results indicated that IFNγ exerts an inhibitory effect on Klotho protein expression in MES-13 cells.

### Recombinant Klotho protein inhibited IFNγ-induced SAMHD1 expression by blocking IFNγ-induced NFκB nuclear translocation in MES-13 cells

**Figure 3:**
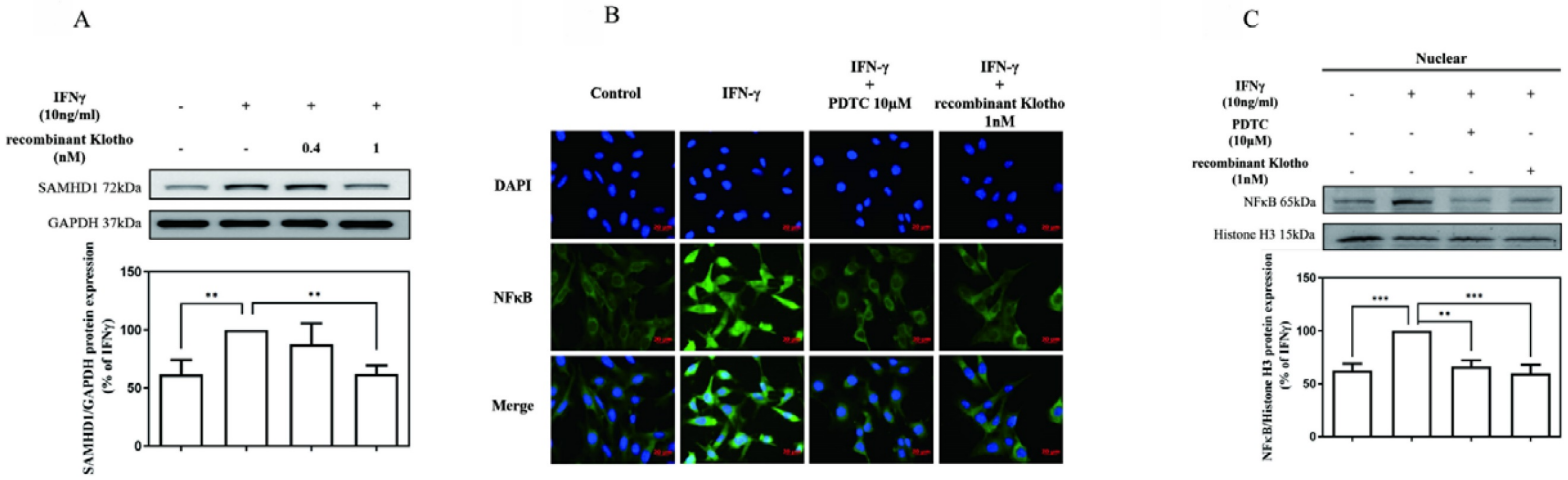
** Recombinant Klotho protein inhibited IFNγ-induced SAMHD1 protein expression in MES-13 cells.** In (A), MES-13 cells were pretreated with recombinant Klotho protein (0.4 nM, 1 nM) for 24 h and then stimulated with 10 ng/mL IFNγ for 24 h. The SAMHD1 protein level in the IFNγ (10 ng/mL)-treated MES-13 cells was used as the control value and set to 100%. The relative protein levels were quantified through scanning densitometry and are expressed as a percentage of the maximal band intensity of the SAMHD1 protein from cultures treated with IFNγ (10 ng/mL). Data are presented as the mean ± SD of Klotho/GAPDH from at least three separate experiments. Asterisks indicate a significant difference from treatment without IFNγ. In (B), the effect of recombinant Klotho protein and PDTC on NFκB nuclear translocation in IFNγ-activated MES-13 cells was visualized through immunofluorescence microscopy. MES-13 cells were pretreated with recombinant Klotho protein (1 nM) for 24 h or with NFκB inhibitor PDTC (10 μM) for 30 min and then stimulated with 10 ng/mL IFNγ for 1.5 h. After fixation on slide glasses, nuclei of MES-13 cells were stained with DAPI to label nuclear DNA (top panels, blue signal). Samples were also stained with an NFκB antibody, and this was followed by signal amplification with a fluorescein isothiocyanate-conjugated secondary antibody (middle panels, green signal). The bottom panel is a merged image of the two upper panels. In (C), the levels of NFκB p65 in nuclear extracts were prepared by nuclear and cytoplasmic extraction as described in methods. Western blots were performed to detect the NFκB distribution in nuclear fractions. The protein amount was quantified by measuring the NFκB bands using densitometric analysis with AlphaEaseFC (Genetic Technologies, Miami, FL, USA) and normalized to histone H3 (H3). All experiments were performed at least in triplicate and reported as a percentage of the control. Data are presented as the mean ± SD of NFκB/H3 derived from at least three separate experiments. Asterisks indicate a significant difference from treatment with IFNγ (10 ng/mL) (* *p* < 0.05, ** *p* < 0.01, *** *p* < 0.001).

To further investigate whether exogenously supplied Klotho modulated IFNγ-induced SAMHD1 expression, MES-13 cells were pretreated with recombinant Klotho protein (0.4 nM, 1 nM) for 24 h and then stimulated with 10 ng/mL IFNγ for 24 h. As illustrated in **Fig. 3 (A**), pretreatment of cells with 1 nM recombinant Klotho protein attenuated IFNγ-induced SAMHD1 protein expression significantly. The amount of IFNγ-induced SAMHD1 protein detected in MES-13 cells was only 60% of that detected in the untreated group. To further elucidate the mechanism underlying the inhibitory effect of recombinant Klotho protein on IFNγ-induced SAMHD1 protein expression, the effect of recombinant Klotho protein on NFκB signaling was examined. As illustrated in **Fig. 3 (B)**, the effect of recombinant Klotho protein and PDTC on NFκB nuclear translocation in IFNγ-activated MES-13 cells was visualized through immunofluorescence microscopy. MES-13 cells were pretreated with either recombinant Klotho protein (1 nM) for 24 h or the NFκB inhibitor PDTC (10 μM) for 30 min and then stimulated with 10 ng/mL IFNγ for 1.5 hr. Without treatment, NFκB was mainly localized in cytoplasmic fractions of MES-13 cells. The nuclear translocation of p65 was observed after the addition of IFNγ to MES-13 cells. The presence of either recombinant Klotho protein or PDTC significantly blocked the nuclear translocation of p65 triggered by IFNγ. Also illustrated in **Fig. 3 (C)**, treatment of IFNγ increased the nuclear amount of NFκB p65 in MES-13 cells. Conversely, pretreatment of MES-13 cells with either recombinant Klotho protein or PDTC markedly reduced the IFNγ-induced NFκB nuclear levels. These results suggested that recombinant Klotho protein inhibited IFNγ-induced SAMHD1 expression by blocking IFNγ-induced NFκB nuclear translocation in MES-13 cells.

### Recombinant Klotho protein inhibited IFNγ-induced SAMHD1 expression not through JAK-STAT1 signaling

**Figure 4:**
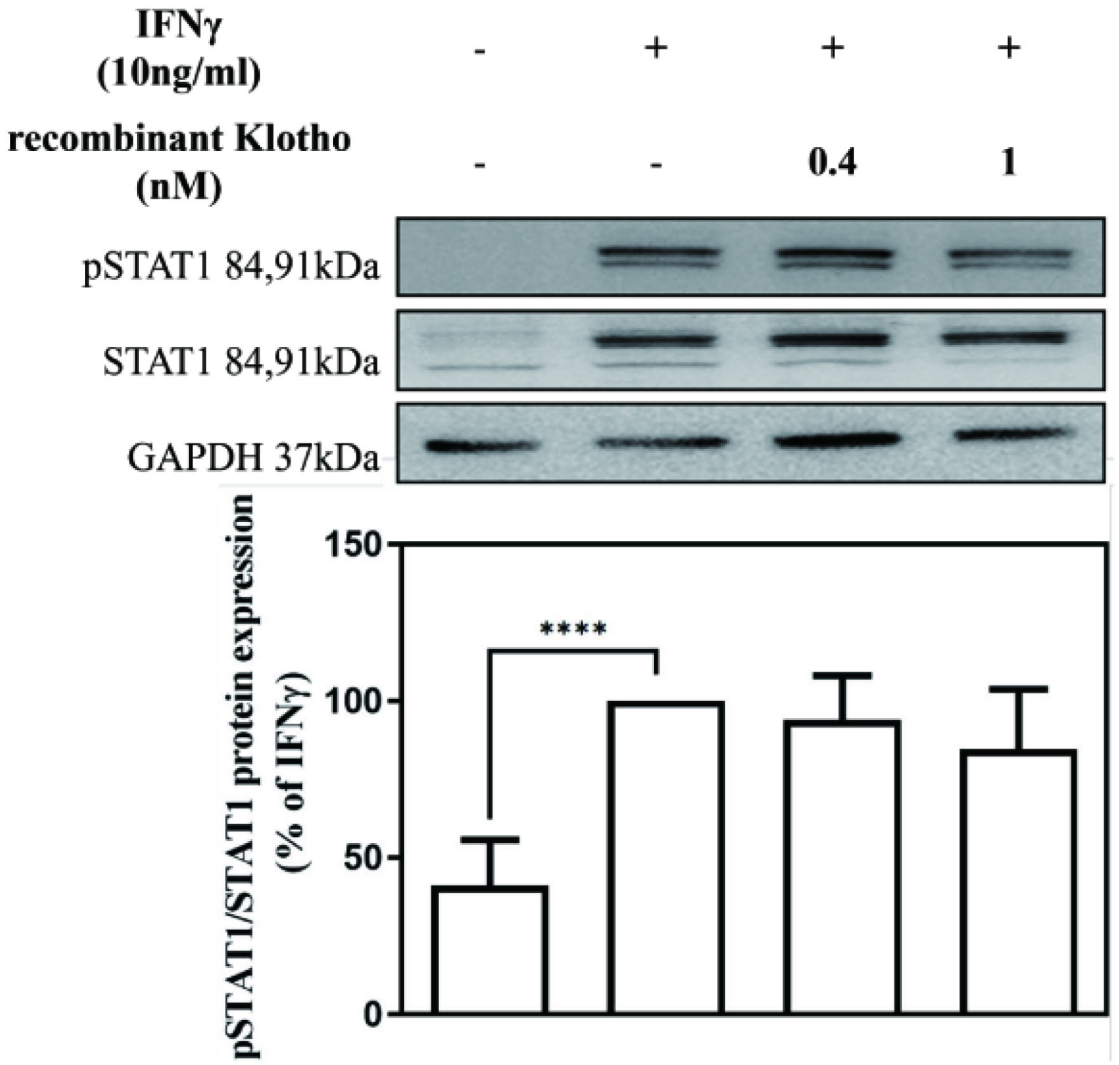
** Recombinant Klotho protein inhibited IFNγ-induced SAMHD1 expression not through JAK-STAT1 signaling.** MES-13 cells were pretreated with recombinant Klotho protein (0.4 nM, 1 nM) for 24 h and then stimulated with 10 ng/mL IFNγ for 1.5 h. The effects of recombinant Klotho protein on IFNγ-induced protein levels of STAT1 and phosphorylated STAT1 (Tyr 701) in MES-13 cells were then determined through Western blot analysis. Phosphorylation of Stat1 at Tyr701 induces transcription factor activation in response to IFNγ. The values of the STAT1 and phosphorylated STAT1 protein levels in IFNγ (10 ng/mL)-treated MES-13 cells were used as controls and set to 100%. Relative protein levels were quantified through scanning densitometry and are expressed as a percentage of the maximal band intensity of the STAT1 and phosphorylated STAT1 proteins from cultures treated with IFNγ (10 ng/mL). Data are presented as the mean ± SD of STAT1 and phosphorylated STAT1 protein/GAPDH derived from at least three separate experiments. Asterisks indicate a significant difference from treatment without IFNγ (∗∗∗∗*p* < 0.0001).

To elucidate the mechanism underlying the inhibitory effect of recombinant Klotho protein on IFNγ-induced SAMHD1 protein expression, the effect of the recombinant Klotho protein on STAT1 signaling was also examined. MES-13 cells were pretreated with recombinant Klotho protein (0.4 nM, 1 nM) for 24 h and then stimulated with 10 ng/mL IFNγ for 1.5 h. The protein level of STAT1 and phosphorylated STAT1 in MES-13 cells was then determined by Western blot. As illustrated in **Fig. 4**, no changes in the protein level of STAT1 and phosphorylated STAT1 were observed in MES-13 cells after recombinant Klotho protein treatment. These results suggested that the inhibitory effect of recombinant Klotho protein on IFNγ-induced SAMHD1 expression was not accomplished through JAK-STAT1 signaling in MES-13 cells.

## Discussion

IFNγ is a major cytokine that profoundly affects the pathogenesis of autoimmune diseases. Increased levels of IFNγ were detected in the peripheral blood mononuclear cells of patients with SLE 24 and in renal biopsy tissue of patients with diffuse proliferative glomerulonephritis 25. The blocking of IFNγ signaling was indicated as a therapeutic strategy for the treatment of SLE 5.

SAMHD1 is an IFNγ-inducible protein that modulates cellular dNTP levels. Mutations of the *SAMHD1* gene have been identified in AG syndrome 26. The clinical phenotypes of AG syndrome are primarily associated with encephalopathy, glaucoma, and chilblains 26. In some cases, juvenile patients with AG syndrome share some similar clinical features with those of SLE 10, 11. Both AG syndrome and SLE are autoimmune diseases, characterized by elevated type I IFN production, intracellular accumulation of nucleic acids due to defects of nucleases, production of autoantibodies, and imbalance in cytokines profiles and signaling 27. AG syndrome can also be caused by mutations in several other intracellular nucleases, such as RNase H2, adenosine deaminase (ADAR1), the RNA sensor melanoma differentiation associated protein 5 (MDA5; encoded by IFN-induced with helicase C domain 1 [*IFIH1*]) and 3′ repair exonuclease (TREX1) 28. Interestingly, mutations of TREX1 and RNase H2 gene were found in SLE patients 29-31 and elevated SAMDH1 gene expression levels were found in peripheral blood from patients with paediatric-onset systemic lupus erythematosus 32. All these finding suggests genetic and pathological connection between AGs, SLE and SAMHD1 protein.

The physiological function of SAMHD1 in kidney is unknown. Although neurologic disorders are major clinical features of AG syndrome, still few cases report patients with AG syndrome developed renal diseases 33, 34. Clinical features include chronic kidney inflammation 34 and collapsing glomerulopathy 33 have been observed in young AG syndrome patients with *RNase H2B* mutation. Moreover, due to the phenotypic overlap between infantile SLE and AG syndrome, and SAMHD1 has been identified as one of the genes implicating in monogenic lupus, a rare form of lupus affecting mainly the kidneys and central nervous system 35, SAMHD1may be possibly involved in kidney diseases such as lupus nephritis. In the childhood-onset form of SLE, a high prevalence of complex clinical symptoms including glomerulonephritis have been reported 36, 37. Mesangial cells* constitute nearly 33% of the cell population within the glomerulus and are major* targets for the renal damage associated with *lupus nephritis 38*. Therefore, in the current study, MES-13 glomerular mesangial cells were used to study the molecular mechanisms underlying IFNγ-induced SAMHD1 gene expression.

Multiple molecular mechanisms including protein phosphorylation 39, promoter methylation 40, and microRNA 41 have been demonstrated to modulate SAMHD1 expression. In the present study, the induction of SAMHD1 by IFNγ was verified in MES-13 cells (**Fig. 1 [A]**). Both nifuroxazide (a STAT1 inhibitor) and PDTC (an effective NFκB inhibitor) reduced IFNγ-induced SAMHD1 expression in MES-13 cells (**Fig. 1 [B-C]**). These data indicate that NFκB is involved in the activation of IFNγ-induced SAMHD1 gene expression in MES-13 cells, despite NFκB not being a canonical transcription factor of IFNγ-targeted genes.

Klotho is an anti-aging protein implicated in the pathogenesis of many human diseases. Decreased Klotho levels were detected in serum from patients with SLE 23. Whether Klotho inhibits nephritis by blocking IFNγ-targeted proteins and IFNγ downstream signaling remains unclear. As illustrated in **Fig. 2**, IFNγ with concentrations above 100 ng/mL significantly decreased Klotho protein expression in MES-13 cells. To further elucidate whether Klotho inhibits nephritis by blocking IFNγ-targeted proteins and IFNγ downstream signaling, MES-13 cells were pretreated with recombinant Klotho protein for 24 h and then stimulated with 10 ng/mL IFNγ for 24 h. As illustrated in **Fig. 3 (A)**, pretreatment of cells with 1 nM recombinant Klotho protein significantly attenuated IFNγ-induced SAMHD1 protein expression. Moreover, as illustrated in **Fig. 3 (B-C)** and **Fig. 4**, recombinant Klotho protein inhibited SAMHD1 expression by blocking IFNγ-induced NFκB nuclear translocation but not JAK-STAT1 signaling in MES-13 cells. These results support the protective role of Klotho in autoimmune disease that can be attributed to its inhibitory effect on IFNγ-induced SAMHD1 expression and IFNγ downstream signaling in MES-13 cells.

In summary, we demonstrated that both SAMHD1 and Klotho were IFNγ-targeted proteins and that recombinant Klotho suppressed IFNγ-induced SAMHD1 expression by blocking nuclear translocation of NFκB in MES-13 cells. Klotho has gained attention because of its emerging role in the treatment of autoimmune diseases, and reduced levels of Klotho were detected in the serum of patients with SLE 23. Our results indicated that the participation of Klotho in lupus nephritis may be partially attributed to its inhibitory effect on IFNγ-induced SAMHD1 expression and NFκB activation in MES-13 cells.

## Abbreviations

ADAR1: adenosine deaminase
AG syndrome: Aicardi-Goutières syndrome
DAPI: 4'-6-diamidino-2-phenylindole
FITC: fluorescein isothiocyanate
GAPDH: glyceraldehyde 3-phosphate dehydrogenase
IFN: interferon
IFNγ: Interferon gamma
IFIH1: IFN-induced with helicase C domain 1
JAK: Janus kinase
MDA5: melanoma differentiation associated protein 5
NFκB: nuclear factor kappa B
PDTC: ammonium pyrrolidinedithiocarbamate
RA: rheumatoid arthritis
SAMHD1: SAM and HD domain-containing protein 1
SDS-PAGE: sodium dodecyl sulfate polyacrylamide gel electrophoresis
STAT: signal transducer and activator of transcription
SLE: systemic lupus erythematosus
TREX1: 3' repair exonuclease

## References

[1] Schroder K, Hertzog PJ, Ravasi T, Hume DA (2004). Interferon-gamma: an overview of signals, mechanisms and functions. Journal of leukocyte biology.

[2] Pollard KM, Cauvi DM, Toomey CB, Morris KV, Kono DH (2013). Interferon-γ and systemic autoimmunity. Discov Med.

[3] Pisetsky DS (2019). The central role of nucleic acids in the pathogenesis of systemic lupus erythematosus. F1000Research.

[4] Anders HJ, Saxena R, Zhao MH, Parodis I, Salmon JE, Mohan C (2020). Lupus nephritis. Nature reviews Disease primers.

[5] Ozmen L, Roman D, Fountoulakis M, Schmid G, Ryffel B, Garotta G (1995). Experimental therapy of systemic lupus erythematosus: the treatment of NZB/W mice with mouse soluble interferon-gamma receptor inhibits the onset of glomerulonephritis. European journal of immunology.

[6] Lafuse WP, Brown D, Castle L, Zwilling BS (1995). Cloning and characterization of a novel cDNA that is IFN-gamma-induced in mouse peritoneal macrophages and encodes a putative GTP-binding protein. Journal of leukocyte biology.

[7] Coggins SA, Mahboubi B, Schinazi RF, Kim B (2020). SAMHD1 Functions and Human Diseases. Viruses.

[8] Crow YJ, Rehwinkel J (2009). Aicardi-Goutieres syndrome and related phenotypes: linking nucleic acid metabolism with autoimmunity. Human molecular genetics.

[9] Ramantani G, Häusler M, Niggemann P, Wessling B, Guttmann H, Mull M (2011). Aicardi-Goutières syndrome and systemic lupus erythematosus (SLE) in a 12-year-old boy with SAMHD1 mutations. J Child Neurol.

[10] Dale RC, Tang SP, Heckmatt JZ, Tatnall FM (2000). Familial systemic lupus erythematosus and congenital infection-like syndrome. Neuropediatrics.

[11] De Laet C, Goyens P, Christophe C, Ferster A, Mascart F, Dan B (2005). Phenotypic overlap between infantile systemic lupus erythematosus and Aicardi-Goutières syndrome. Neuropediatrics.

[12] Jin C, Peng X, Liu F, Cheng L, Xie T, Lu X (2016). Interferon-induced sterile alpha motif and histidine/aspartic acid domain-containing protein 1 expression in astrocytes and microglia is mediated by microRNA-181a. AIDS (London, England).

[13] Schmidt S, Schenkova K, Adam T, Erikson E, Lehmann-Koch J, Sertel S (2015). SAMHD1's protein expression profile in humans. Journal of leukocyte biology.

[14] Abe T, Fleming PA, Masuya M, Minamiguchi H, Ebihara Y, Drake CJ (2005). Granulocyte/macrophage origin of glomerular mesangial cells. International journal of hematology.

[15] Masuya M, Drake CJ, Fleming PA, Reilly CM, Zeng H, Hill WD (2003). Hematopoietic origin of glomerular mesangial cells. Blood.

[16] Schlöndorff D, Banas B (2009). The Mesangial Cell Revisited: No Cell Is an Island. Journal of the American Society of Nephrology.

[17] Morales E, Galindo M, Trujillo H, Praga M (2021). Update on Lupus Nephritis: Looking for a New Vision. Nephron.

[18] Kurosu H, Yamamoto M, Clark JD, Pastor JV, Nandi A, Gurnani P (2005). Suppression of aging in mice by the hormone Klotho. Science (New York, NY).

[19] Xu Y, Sun Z (2015). Molecular basis of Klotho: from gene to function in aging. Endocrine reviews.

[20] Thurston RD, Larmonier CB, Majewski PM, Ramalingam R, Midura-Kiela M, Laubitz D (2010). Tumor necrosis factor and interferon-gamma down-regulate Klotho in mice with colitis. Gastroenterology.

[21] Koh N, Fujimori T, Nishiguchi S, Tamori A, Shiomi S, Nakatani T (2001). Severely reduced production of klotho in human chronic renal failure kidney. Biochemical and biophysical research communications.

[22] Witkowski JM, Soroczyńska-Cybula M, Bryl E, Smoleńska Ż, Jóźwik A (2007). Klotho—a Common Link in Physiological and Rheumatoid Arthritis-Related Aging of Human CD4^+^ Lymphocytes. The Journal of Immunology.

[23] Russell DL, Oates JC, Markiewicz M (2021). Association Between the Anti-Aging Gene Klotho and Selected Rheumatologic Autoimmune Diseases. The American journal of the medical sciences.

[24] Harigai M, Kawamoto M, Hara M, Kubota T, Kamatani N, Miyasaka N (2008). Excessive production of IFN-gamma in patients with systemic lupus erythematosus and its contribution to induction of B lymphocyte stimulator/B cell-activating factor/TNF ligand superfamily-13B. Journal of immunology (Baltimore, Md: 1950).

[25] Masutani K, Akahoshi M, Tsuruya K, Tokumoto M, Ninomiya T, Kohsaka T (2001). Predominance of Th1 immune response in diffuse proliferative lupus nephritis. Arthritis and rheumatism.

[26] Crow YJ, Manel N (2015). Aicardi-Goutières syndrome and the type I interferonopathies. Nature reviews Immunology.

[27] Wang L, Wang F-S, Gershwin ME (2015). Human autoimmune diseases: a comprehensive update. Journal of Internal Medicine.

[28] Behrendt R, Schumann T, Gerbaulet A, Nguyen LA, Schubert N, Alexopoulou D (2013). Mouse SAMHD1 has antiretroviral activity and suppresses a spontaneous cell-intrinsic antiviral response. Cell Rep.

[29] Pendergraft WF 3rd, Means TK (2015). AGS, SLE, and RNASEH2 mutations: translating insights into therapeutic advances. The Journal of clinical investigation.

[30] Günther C, Kind B, Reijns MA, Berndt N, Martinez-Bueno M, Wolf C (2015). Defective removal of ribonucleotides from DNA promotes systemic autoimmunity. The Journal of clinical investigation.

[31] Lee-Kirsch MA, Gong M, Chowdhury D, Senenko L, Engel K, Lee YA (2007). Mutations in the gene encoding the 3'-5' DNA exonuclease TREX1 are associated with systemic lupus erythematosus. Nat Genet.

[32] Zhao X, Li C, Li S, Zhang J, Kuang W, Deng J (2022). SAMHD1 associates with inflammation and vasculitis in paediatric-onset systemic lupus erythematosus. Clinical and experimental rheumatology.

[33] Fenaroli P, Rossi GM, Angelotti ML, Antonelli G, Volpi S, Grossi A (2021). Collapsing Glomerulopathy as a Complication of Type I Interferon-Mediated Glomerulopathy in a Patient With RNASEH2B-Related Aicardi-Goutières Syndrome. American journal of kidney diseases: the official journal of the National Kidney Foundation.

[34] He T, Xia Y, Yang J (2021). Systemic inflammation and chronic kidney disease in a patient due to the RNASEH2B defect. Pediatr Rheumatol Online J.

[35] Alperin JM, Ortiz-Fernández L, Sawalha AH (2018). Monogenic Lupus: A Developing Paradigm of Disease. Front Immunol.

[36] Webb R, Kelly JA, Somers EC, Hughes T, Kaufman KM, Sanchez E (2011). Early disease onset is predicted by a higher genetic risk for lupus and is associated with a more severe phenotype in lupus patients. Ann Rheum Dis.

[37] Webber D, Cao J, Dominguez D, Gladman DD, Levy DM, Ng L (2020). Association of systemic lupus erythematosus (SLE) genetic susceptibility loci with lupus nephritis in childhood-onset and adult-onset SLE. Rheumatology (Oxford, England).

[38] Wright RD, Dimou P, Northey SJ, Beresford MW (2019). Mesangial cells are key contributors to the fibrotic damage seen in the lupus nephritis glomerulus. Journal of Inflammation.

[39] Batalis S, Rogers LC, Hemphill WO, Mauney CH, Ornelles DA, Hollis T (2021). SAMHD1 Phosphorylation at T592 Regulates Cellular Localization and S-phase Progression. Front Mol Biosci.

[40] de Silva S, Hoy H, Hake TS, Wong HK, Porcu P, Wu L (2013). Promoter methylation regulates SAMHD1 gene expression in human CD4+ T cells. The Journal of biological chemistry.

[41] Kohnken R, Kodigepalli KM, Mishra A, Porcu P, Wu L (2017). MicroRNA-181 contributes to downregulation of SAMHD1 expression in CD4+ T-cells derived from Sezary syndrome patients. Leukemia research.

